# Task Order Moderates the Effects of APOE Genotype Awareness on Digit Span Performance in Cognitively Unimpaired Older Adults

**DOI:** 10.1002/alz70857_105056

**Published:** 2025-12-25

**Authors:** Meenakshi Menon, Katherine J Shoemaker, Mara Mather, Jason Karlawish, Jessica B. Langbaum, Sarah Barber

**Affiliations:** ^1^ Georgia State University, Atlanta, GA, USA; ^2^ University of Southern California, Leonard Davis School of Gerontology, Los Angeles, CA, USA; ^3^ University of Pennsylvania Perelman School of Medicine, Philadelphia, PA, USA; ^4^ Banner Alzheimer's Institute, Phoenix, AZ, USA

## Abstract

**Background:**

As the prevalence of APOE testing rises, it is crucial to understand how APOE genotype and genotype awareness impact cognitive performance. This study investigates the effects of APOE genotype, genotype awareness, and task order on digit span performance in cognitively unimpaired older adults.

**Method:**

Of the 196 participants (M_age_ = 71.14, range = 63–79), 69.4% were APOE ε4 carriers. A total of 69.1% of ε4 carriers and 53.3% of ε4 non‐carriers were aware of their genotype. Participants were told the study aimed to examine the effects of APOE genotype on cognitive performance. After receiving this instruction participants completed a randomized battery of cognitive tests, including Forward and Backward Digit Span (FDS, BDS). The primary analysis was a MANOVA examining the effects of APOE genotype, and genotype awareness, on FDS and BDS performance. An exploratory analysis assessed whether the results varied based on task order.

**Result:**

In the primary analysis there were no significant results (ps > .174). However, adding task order as a factor resulted in a significant interaction between genotype, genotype awareness, and task order for BDS (*p* = .026). Follow‐up 2(genotype awareness) X 2(task order) ANOVAs on BDS revealed a significant interaction between these factors for ε4 carriers (*p* = .002) but not for ε4 non‐carriers (*p* = .552). Among the ε4 carriers, knowledge of genotype was associated with numerically poorer performance when digit span tasks were completed first (Aware: M = 8.69, Unaware: M = 10.69; *p* = .052) but significantly better performance when digit span tasks were completed last (Aware: M = 10.35, Unaware: M = 7.56; *p* = .016).

**Conclusion:**

Among APOE ε4 carriers the effects of genotype awareness on BDS performance varied based upon whether the digit span tasks were completed first or last within the cognitive battery. Among ε4 carriers who performed the digit span tasks first, genotype awareness was linked to lower BDS performance, possibly due to stereotype threat. This effect reversed for among ε4 carriers who completed the digit span tasks last. These findings align with research suggesting stronger threat effects when the dependent variable is measured soon after the manipulation (Lamont et al., 2015).

